# Growth differentiation factor-15 and the association between type 2 diabetes and liver fibrosis in NAFLD

**DOI:** 10.1038/s41387-021-00170-3

**Published:** 2021-10-18

**Authors:** Josh Bilson, Eleonora Scorletti, Laure B. Bindels, Paul R. Afolabi, Giovanni Targher, Philip C. Calder, Jaswinder K. Sethi, Christopher D. Byrne

**Affiliations:** 1 Human Development and Health, Faculty of Medicine, University of Southampton, Southampton, UK; 2 National Institute for Health Research Southampton Biomedical Research Centre, University of Southampton and University Hospital Southampton National Health Service Foundation Trust, Southampton, UK; 3 Division of Gastroenterology, Department of Medicine, Perelman School of Medicine, University of Pennsylvania, Philadelphia, PA 19104 USA; 4 Metabolism and Nutrition Research Group, Louvain Drug Research Institute, Université Catholique de Louvain, Brussels, Belgium; 5 Section of Endocrinology, Diabetes and Metabolism, Department of Medicine, University and Azienda Ospedaliera Universitaria Integrata of Verona, Verona, Italy; 6 Institute for Life Sciences, University of Southampton, Southampton, UK

**Keywords:** Obesity, Risk factors, Type 2 diabetes

## Abstract

**Background:**

Type 2 diabetes mellitus (T2DM) is a strong risk factor for liver fibrosis in non-alcoholic fatty liver disease (NAFLD). It remains uncertain why T2DM increases the risk of liver fibrosis. It has been suggested that growth differentiation factor-15 (GDF-15) concentrations increase the risk of liver fibrosis. We aimed to investigate (a) whether GDF-15 concentrations were associated with liver fibrosis and involved in the relationship between T2DM and liver fibrosis and (b) what factors linked with T2DM are associated with increased GDF-15 concentrations.

**Methods:**

Ninety-nine patients with NAFLD (61% men, 42.4% T2DM) were studied. Serum GDF-15 concentrations were measured by electro-chemiluminescence immunoassay. Vibration-controlled transient elastography (VCTE)-validated thresholds were used to assess liver fibrosis. Regression modelling, receiver operator characteristic curve analysis and Sobel test statistics were used to test associations, risk predictors and the involvement of GDF-15 in the relationship between T2DM and liver fibrosis, respectively.

**Results:**

Patients with NAFLD and T2DM (*n* = 42) had higher serum GDF-15 concentrations [mean (SD): 1271.0 (902.1) vs. 640.3 (332.5) pg/ml, *p* < 0.0001], and a higher proportion had VCTE assessed ≥F2 fibrosis (48.8 vs. 23.2%, *p* = 0.01) than those without T2DM. GDF-15 was independently associated with liver fibrosis (*p* = 0.001), and GDF-15 was the most important single factor predicting ≥F2 or ≥F3 fibrosis (≥F2 fibrosis AUROC 0.75, (95% CI 0.63–0.86), *p* < 0.001, with sensitivity, specificity, positive predictive (PPV) and negative predictive (NPV) values of 56.3%, 86.9%, 69.2% and 79.1%, respectively). GDF-15 was involved in the association between T2DM and ≥F2 fibrosis (Sobel test statistic 2.90, *p* = 0.004). Other factors associated with T2DM explained 60% of the variance in GDF-15 concentrations (*p* < 0.0001). HbA1c concentrations alone explained 30% of the variance (*p* < 0.0001).

**Conclusions:**

GDF-15 concentrations are a predictor of liver fibrosis and potentially involved in the association between T2DM and liver fibrosis in NAFLD. HbA1c concentrations explain a large proportion of the variance in GDF-15 concentrations.

## Introduction

Non-alcoholic liver disease (NAFLD) is a ‘multisystem’ disease that increases the risk of type 2 diabetes mellitus (T2DM), cardiovascular disease (CVD) [1], and chronic kidney disease (CKD) [2]. Bi-directional relationships exist between NAFLD and T2DM and not only is NAFLD an independent risk factor for incident T2DM, but when both diseases co-exist, T2DM increases the risk of faster progression of NAFLD to advanced fibrosis, cirrhosis and hepatocellular carcinoma [2–4]. However, the factors involved in the association between T2DM and increased risk of liver fibrosis in patients with NAFLD are not fully understood.

Growth differentiation factor-15 (GDF-15), also known as macrophage inhibitory cytokine (MIC)-1, is a stress-inducible cytokine that can be ubiquitously expressed [5]. Circulating GDF-15 concentrations are increased in patients with T2DM [6, 7], and are separately reported to associate with obesity [8], liver disease severity [9, 10], CVD [11] and CKD [12]. A recent multicentre transcriptomic study demonstrated that hepatic GDF-15 expression was positively associated with NAFLD severity and GDF-15 expression was significantly higher in patients with advanced liver fibrosis [10]. In support of these findings, a previous study measured serum GDF-15 concentrations in patients (in an Asian population) with both T2DM and NAFLD and showed that GDF-15 concentrations were higher in those with T2DM and advanced liver fibrosis [13]. Furthermore, growing evidence suggests that GDF-15 may have pro-fibrogenic effects within the liver and other tissues [13–15]. Taken together, these studies suggest that increased GDF-15 concentrations may increase the risk of liver fibrosis. However, it is not known whether circulating GDF-15 concentrations are potentially involved in the known relationship between T2DM and liver fibrosis in patients with NAFLD.

Additional factors associated with T2DM have also been proposed to explain why T2DM is a risk factor for liver fibrosis. These include insulin resistance, altered adipokine concentrations [16] and altered gut microbiota composition [17]. Moreover, it has also been suggested that oral hypoglycaemic agents, such as metformin, can affect circulating GDF-15 concentrations [18, 19]. Whether these factors explain the increase in GDF-15 concentration in patients with T2DM and NAFLD remains uncertain. Therefore we aimed to test (a) whether GDF-15 concentrations were a predictor of liver fibrosis and potentially involved in the association between T2DM and liver fibrosis and (b) what factors linked with T2DM are independently associated with, and explain the variance in GDF-15 concentrations, in patients with NAFLD.

## Materials and methods

A total of 99 predominantly Northern European patients with NAFLD (age range of 20–77 years) were studied to perform this secondary analysis of baseline characteristics of patients recruited to the INSYTE (**In**vestigation of **Sy**nbiotic **T**reatm**e**nt in NAFLD) trial (www.clinicaltrials.gov registered number NCT01680640). These patients were recruited as described in detail previously [20, 21]. The trial design was approved by the Southampton and South West Hampshire research ethics committee (12/SC/0614). All patients gave their written informed consent.

### Inclusion and exclusion criteria

The inclusion and exclusion criteria for the INSYTE trial have been previously described in detail [20, 21]. Briefly, participants were aged >18 years with a diagnosis of NAFLD confirmed in secondary care, with evidence of hepatic steatosis confirmed by via proton magnetic resonance spectroscopy (^1^H-MRS) at recruitment.

### Anthropometric and biochemical measurements

Anthropometric and biochemical measurements were collected as previously described [20, 21]. Body composition was assessed by dual-energy X-ray absorptiometry (DEXA). Blood pressure was measured using a Marquette Dash 300 monitor (GE Healthcare, Little Chalfont, Bucks, UK) as previously described [21]. Handgrip strength was measured using a Jamar hand Dynamometer with participants seated and their arms rested on the chair arms—data are presented as grip strength (kg). Fasting glucose, haemoglobin A1c (HbA1c), fasting insulin, total cholesterol, high-density lipoprotein (HDL)-cholesterol, triglycerides, alanine aminotransferase (ALT), aspartate aminotransferase (AST), adiponectin, leptin, high-sensitivity C-reactive protein (hs-CRP), tumour necrosis factor-α (TNFα), interleukin (IL)−6, IL-8 and IL-10 concentrations were measured in serum samples using commercially available kits according to the manufacturer’s instructions. Serum lipopolysaccharide (LPS) concentrations were measured as previously described [20]. Concentrations of GDF-15, leptin and adiponectin were measured in fasting serum samples by the Cambridge Biochemical Assay Laboratory, University of Cambridge. Serum GDF-15 quantification was done with antibodies and standards from R&D Systems (R&D Systems - catalogue number DY957) and in accordance with manufacturer’s instructions and as previously described [18]. The estimated glomerular filtration rate (e-GFR) was measured using the CKD-Epidemiology Collaboration (CKD-EPI) study equation [22]. Satiety hormones, such as plasma ghrelin, gastric inhibitory polypeptide (GIP), glucagon-like peptide (GLP-1), peptide YY (PYY) and pancreatic peptide (PP) concentrations were also measured as previously described [21] using the MILLIPLEX®MAP Human Metabolic Hormone Panels Kit.

### Liver fat and vibration-controlled transient elastography measurements

Liver fat and VCTE-derived kPa measurements were collected as previously described [20, 21]. In all participants, the quantification of intra-hepatic fat content was undertaken via proton magnetic resonance spectroscopy (^1^H-MRS) (see Supplementary Material for method). Liver VCTE-derived kPa measurements were assessed as a clinically recognised proxy measure of liver elasticity using the Echosens (Waltham, MA) Fibroscan® by a trained clinician (ES). Results are expressed as the medians (IQRs) in kilo-pascals (kPa). Liver VCTE-derived kPa measurements of ≥8.2 kPa and ≥9.7 kPa were used as validated proxy thresholds for identification ≥F2 and ≥F3 fibrosis with the former having a AUROC of 0.77 (95% CI 0.72, 0.82) for the prediction of ≥F2 fibrosis (sensitivity and specificity = 71% and 70%, respectively) as recently reported [23]. The technical description and examination procedures for liver VCTE-kPa measurements have also been previously described [24].

### Appetite, hunger and satiety assessment

Assessment of patient appetite, hunger and satiety was done as previously described [20, 21]. See Supplementary Material for further description of this methodology.

### Gut microbiota analyses—DNA extraction, sequencing and bioinformatics

Gut microbiota DNA extraction from faecal samples, 16S amplicon sequencing and bioinformatic analyses were performed as previously described [20, 21]. See Supplementary Material for further details of this methodology.

### Statistical analysis

Data were analysed using Statistical Package for the Social Sciences (SPSS) Version 26.0 (New York, USA). Data were tested for normality using the Shapiro–Wilk and Kolmogorov–Smirnov tests and are presented as means (SD) for normally distributed variables and medians and inter-quartile ranges (IQRs) for non-normally distributed variables. Comparisons of continuous variables between groups were performed with the unpaired Student *t*-test for normally distributed variables and the Mann–Whitney *U* test for non-normally distributed variables, and differences in proportions were investigated using the chi-squared test or the Fisher’s exact test as appropriate. Univariable associations between variables were investigated using Pearson’s correlation for normally distributed or Spearman’s rank correlation for non-normally distributed variables. To test for the independence of associations between explanatory factors and serum GDF-15 concentrations, VCTE-measured liver kPa measurements or additional liver fibrosis biomarkers, factors were entered into a multivariable linear regression model with either: (a) logarithmically transformed GDF-15 concentrations; (b) logarithmically transformed liver kPa measurements; (c) Enhanced liver fibrosis (ELF) scores; (d) logarithmically transformed Fibrosis-4 (FIB-4) scores; (e) logarithmically transformed AST to platelet ratio index (APRI) scores or (f) hepatic mitochondrial function (HMF) (as determined by the 13C-ketoisocaproate breath test [13C-KICA BT]) as the outcome variable. Regression models were run with all explanatory factors, or stepwise, to investigate the proportion of total variance in serum GDF-15 concentrations that could be explained by each individual explanatory factor. Binary logistic regression modelling was used to investigate whether serum GDF-15 concentrations and/or T2DM status were independently associated with ≥F2 and ≥F3 fibrosis (as indicated by the validated VCTE measurement of ≥8.2 and ≥9.7 kPa, respectively), to identify whether other liver fibrosis biomarkers were associated with ≥F2 and ≥F3 fibrosis and also to identify factors that were independently associated with high (≥1193.7 pg/ml) serum GDF-15 concentrations. Goodness of fit for the models was tested with Hosmer–Lemeshow tests. Sobel test statistics and *p*-values were calculated to test the potential involvement of GDF-15 concentrations in the association between T2DM and either ≥F2, or ≥F3 fibrosis separately according to [25]. Receiver-operating characteristic (ROC) curve analysis for GDF-15 or HbA1c was performed to estimate areas under the receiver-operating characteristic curves (AUROCs), as well as to estimate the best cut-off values (Youden’s index) to predict ≥F2 and ≥F3 fibrosis, or high serum GDF-15 concentrations. See Supplementary Material for methods used for the statistical analysis of the gut microbiota. Where data were not available on all 99 participants, the number of subjects included in the analysis for which there was complete data are presented in the relevant table or figure legend.

## Results

### Characteristics of participants

**Fig. 1:**
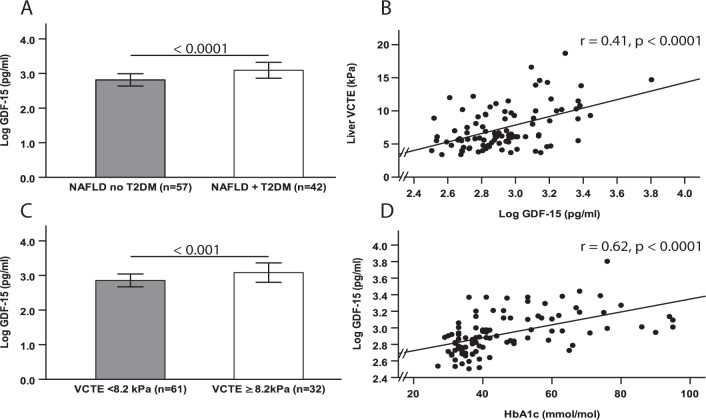
Differences in serum GDF-15 concentrations by type 2 diabetes status, predicted liver fibrosis severity and scatter plots showing the association between serum GDF-15 concentrations and both haemoglobin A1c and liver stiffness measurements (as assessed by vibration-controlled transient elastography [VCTE]). **A** The differences in serum GDF-15 concentrations (logarithmically transformed) between NAFLD patients with and without coexisting type 2 diabetes. **B** The scatter plot for the association of serum GDF-15 concentrations with liver VCTE measurements (kPa). **C** The differences in serum GDF-15 concentrations between NAFLD patients with <F2 or ≥F2 fibrosis according to the validated VCTE measurement threshold of ≥ 8.2 kPa as a proxy for the identification of ≥F2 fibrosis. **D** The scatter plot for the association of serum GDF-15 with HbA1c concentrations. Data are presented as mean ± SD. Associations are Spearman’s rank correlation coefficients. Sample size **A**, **D** *n* = 99; **B**, **C** *n* = 93. VCTE vibration-controlled transient elastography, GDF-15 growth differentiation factor-15, T2DM type 2 diabetes mellitus.

The mean (SD) age of the 99 patients with NAFLD (61 men, 38 women) included in the study was 50.9 (12.8) years. Table 1 shows the characteristics of patients, stratified by T2DM status. In patients with NAFLD and T2DM, liver VCTE-derived kPa measurements were significantly higher and there was a greater prevalence of ≥F2 and ≥F3 liver fibrosis, (according to the previously validated VCTE thresholds of ≥8.2 kPa and ≥9.7 kPa, respectively) compared to counterparts without T2DM. Furthermore, FIB-4 scores were significantly higher in patients with both NAFLD and T2DM. Fasting glucose and HbA1c concentrations were also higher in patients with NAFLD and T2DM, whereas fasting insulin concentrations were not different between groups. Twenty-nine (69%) patients with T2DM were receiving metformin treatment. In patients with NAFLD and T2DM, age, HOMA-IR, IL-8 and hs-CRP were also higher than in those without T2DM whereas HMF was lower in patients with both NAFLD and T2DM. Notably, serum GDF-15 concentrations were markedly higher in patients with NAFLD and T2DM than in those without T2DM (Table 1 and Fig. 1a). Regarding gut microbiota composition, the relative abundance of the Enterobacteriaceae family was greater in patients with NAFLD and T2DM than in those without T2DM (*p* = 0.001, data not shown). In patients with NAFLD and T2DM, fasting ghrelin concentrations were lower and fasting GLP-1 concentrations were higher, compared to patients without T2DM (Supplementary Table 1). Furthermore, following a breakfast challenge, ghrelin AUC was lower and GLP-1, PP and PYY AUCs were higher in patients with NAFLD and T2DM, than in their counterparts without T2DM (Supplementary Table 1). However, neither fasted nor AUC values, for reported hunger, fullness, and satiety, were significantly different in patients with or without T2DM (Supplementary Table 1).

**Table 1:** Characteristics of patients with NAFLD stratified by pre-existing type 2 diabetes status.

Variables	Without T2DM (*n* = 57)	With T2DM (*n* = 42)	*p*-value
Age (yrs)	48.7 ± 14.2	53.8 ± 10.1	0.04
Sex (male) (*n*,%)^a^	38 (66.7%)	23 (54.8%)	0.23
Smoking history (no) (*n*, %)^a^	53 (93%)	34 (81%)	0.12
Systolic blood pressure (mmHg)	133.7 (19.5)	134.4 (27.5)	0.8
Diastolic blood pressure (mmHg)	74.4 ± 8.3	73.9 ± 10.7	0.82
BMI (kg/m^2^)	32.3 (6.4)	34.9 (6.8)	0.06
DEXA lean body mass (kg)	64.5 ± 13.2	62 ± 10.6	0.25
DEXA total body fat (%)	33.8 (10.2)	36.7 (12.2)	0.22
Handgrip strength (kg)	36.7 (25.8)	31.2 (19.5)	0.05
Fasting glucose (mmol/l)	5.3 (1.0)	8.1 (3.3)	<0.0001
Haemoglobin A1c (mmol/mol)	35.0 (5.5)	59.5 (23)	<0.0001
Fasting insulin (mIU/L)^b^	14.2 (9.7)	13.7 (9.0)	0.94
HOMA-IR^c^	3.5 (2.7)	5.4 (5.1)	<0.001
Metformin use (yes) (*n*, %)^a^	0 (0%)	29 (69%)	<0.0001
Triglycerides (mmol/l)	1.8 (1)	1.8 (1.2)	0.27
Total cholesterol (mmol/l)	5.2 (1.4)	4.4 (1.3)	0.001
HDL cholesterol (mmol/l)	1.2 (0.4)	1.2 (0.3)	0.61
AST (IU/l)	34.0 (22.0)	38.0 (31.5)	0.54
ALT (IU/l)	56.0 (45.0)	59.0 (41.8)	0.8
MRS-measured liver fat (%)	23.7 (34.8)	27.0 (24.1)	0.871
13C-KICA BT (cPDR over 1h-%)	14.5 ± 3.7	12.6 ± 3.2	0.008
Liver VCTE (kPa)^c^	6.0 (3.1)	8.0 (4.8)	0.01
Liver VCTE ≥ 8.2 kPa, (yes) (%)^a^	12 (23.2%)	20 (48.8%)	0.01
Liver VCTE ≥ 9.7 kPa, (yes) (%)^a^	7 (13.5%)	15 (36.6%)	0.009
APRI^c^	0.4 (0.3)	0.4 (0.5)	0.44
FIB-4^c^	0.9 (1.2)	1.2 (1.1)	0.02
ELF^d^	6.9 ± 0.4	7.0 ± 0.3	0.06
GDF-15 (pg/ml)	640.3 (332.5)	1271.0 (902.1)	<0.0001
Adiponectin (μg/ml)	4.9 (2.4)	3.8 (2.6)	0.31
Leptin (ng/ml)^d^	20.0 (32.9)	24.4 (31.2)	0.77
TNFα (pg/ml)^d^	12.9 (5.5)	10.4 (4.1)	0.15
IL-6 (pg/ml)	2.6 (1.6)	2.6 (2.0)	0.26
IL-8 (pg/ml)	13.8 (7.2)	17.8 (10.5)	0.01
IL-10 (pg/ml)	0.8 (0.4)	0.7 (0.4)	0.89
hs-CRP (mg/l)	2.0 (3.0)	4.0 (5.3)	0.003
LPS (EU/ml)	0.2 (0.1)	0.1 (0.1)	0.51
e-GFR (ml/min/1.73 m^2^)	90.0 (12.3)	90.0 (9.8)	0.68

Data presented as means ± SDs or medians (inter-quartile ranges).

*T2DM* Type 2 diabetes mellitus, *BMI* body mass index, *DEXA* dual-energy X-ray absorptiometry, *HOMA-IR* homeostatic model assessment of insulin resistance, *HDL* high-density lipoprotein, *AST* aspartate aminotransferase, *ALT* alanine transaminase, *MRS* magnetic resonance spectroscopy, *13C-KICA BT* 13C-ketoisocaproate breath test, *VCTE* vibration-controlled transient elastography, *APRI* AST to platelet ratio index, *FIB-4* Fibrosis-4, *ELF* *enhanced liver fibrosis GDF-15* growth differentiation factor-15, *TNFα* tumour necrosis factor-α, *IL* interleukin, *hs-CRP* high-sensitivity C-reactive protein, *LPS* lipopolysaccharide, *e-GFR* estimated glomerular filtration rate.

^a^Cross-tab. Pearson chi-squared test.

^b^Data were only available for no T2DM *n* = 52 and T2DM *n* = 32.

^c^Data were only available for no T2DM *n* = 52 and T2DM *n* = 41.

^d^Data were only available for no T2DM *n* = 50 and T2DM *n* = 38.

### Serum GDF-15 concentrations predict liver fibrosis and are potentially involved in the association between T2DM and liver fibrosis

In univariable analysis, higher serum GDF-15 concentrations were associated with higher liver VCTE-derived kPa measurements (Fig. 1b), and serum GDF-15 concentrations were higher in patients with ≥F2 fibrosis (Fig. 1c). Both APRI and FIB-4 test scores were positively associated with the presence of ≥F2 and ≥F3 fibrosis independently of age and sex (Supplementary Table 2). Similarly, serum GDF-15 was significantly and positively associated with APRI, FIB-4 and ELF test scores (Table 2). Serum GDF-15 concentrations were associated with higher concentrations of fasting glucose (Table 2) and HbA1c (Fig. 1d) (*r* = 0.60, and *r* = 0.62, respectively, *p* < 0.00001 for both). Serum GDF-15 concentrations were also positively associated with age, BMI, total body fat percentage, HOMA-IR, AST, IL-6, IL-8, hs-CRP concentrations, and negatively with e-GFR, HMF and handgrip strength (Table 2).

**Table 2:** Univariable linear associations with serum GDF-15 concentrations.

Variables	Correlation coefficient(s)	*p*-value
Age (yrs)	0.44	<0.00001
Systolic blood pressure (mmHg)	0.05	0.65
Diastolic blood pressure (mmHg)	0.06	0.56
BMI (kg/m^2^)^a^	0.29	0.003
DEXA lean body mass (kg)	−0.15	0.16
DEXA total body fat (%)	0.22	0.03
Handgrip strength (kg)	−0.31	0.002
Fasting glucose (mmol/l)^a^	0.60	<0.00001
Haemoglobin A1c (mmol/mol)^a^	0.62	<0.00001
Fasting insulin (mIU/L)^b^	0.11	0.32
HOMA-IR^b^	0.41	<0.0001
Triglycerides (mmol/l)^a^	0.20	0.84
Total cholesterol (mmol/l)^a^	−0.29	0.003
HDL cholesterol (mmol/l)^a^	−0.01	0.94
AST (IU/l)^a^	0.29	0.003
ALT (IU/l)^a^	0.13	0.19
MRS-measured liver fat (%)^a^	−0.10	0.35
13C-KICA BT (cPDR over 1h-%)^a^	−0.38	<0.001
Liver VCTE (kPa)^a,c^	0.41	<0.0001
APRI^a,c^	0.28	0.007
FIB-4^a,c^	0.53	<0.00001
ELF^a,d^	0.53	<0.00001
Adiponectin (μg/ml)^d^	−0.02	0.85
Leptin (ng/ml)^d^	0.16	0.14
TNFα (pg/ml)^a^	0.06	0.57
IL-6 (pg/ml)^a^	0.25	0.02
IL-8 (pg/ml)^a^	0.33	0.001
IL-10 (pg/ml)^a^	0.19	0.07
hs-CRP (mg/l)^a^	0.32	0.001
LPS (EU/ml)	0.12	0.25
e-GFR (ml/min/1.73 m^2^)^a^	−0.24	0.018

Sample size, *n* = 99.

*BMI* body mass index, *DEXA* dual-energy X-ray absorptiometry, *HDL* high-density lipoprotein, *AST* aspartate aminotransferase, *ALT* alanine transaminase, *MRS* magnetic resonance spectroscopy, *13C-KICA BT* 13C-ketoisocaproate breath test, *VCTE* vibration-controlled transient elastography, *APRI* AST to platelet ratio index, *FIB-4* Fibrosis-4, *ELF* enhanced liver fibrosis, *GDF-15* growth differentiation factor-15, *TNFα* tumour necrosis factor-α, *IL* interleukin, *hs-CRP* high-sensitivity C-reactive protein, *e-GFR* estimated glomerular filtration rate.

^a^Spearman’s rank correlation coefficients.

^b^Sample size 82.

^c^Samples size *n* = 93.

^d^Sample size 88.

Multivariable linear regression modelling was undertaken to investigate which factors were independently associated with liver VCTE-derived kPa measurements as the outcome variable. In a regression model where GDF-15, age, sex, total body fat, T2DM status, e-GFR and AST were entered as putative key explanatory variables and liver kPa measurement as the outcome, only serum GDF-15 concentrations were independently associated with liver kPa measurement [unstandardised *β* coefficient = 0.35 (95% CI 0.15–0.56), *p* = 0.001 (model fit *R*^2^ = 0.261; *p* = 0.001)] (Supplementary Table 3). In this regression model, GDF-15 concentrations alone explained 21% of the total variance in liver VCTE-derived kPa value. Intriguingly, upon removal of GDF-15 from this model, T2DM status became independently associated with liver VCTE-derived kPa measurement [*β* coefficient = 0.09 (0.18–0.17), *p* = 0.015], whereas age, sex, total body fat, e-GFR and AST were not associated with liver VCTE-derived kPa measurement (model fit *R*^2^ = 0.152; *p* = 0.03). Serum GDF-15 concentrations were also positively and independently associated with ELF, FIB-4 and APRI scores and, according to stepwise analysis, contributed the most towards the total variance of each liver fibrosis biomarker (Supplementary Table 3). Serum GDF-15 concentrations were not independently associated with HMF in a model with the same combination of explanatory variables (data not shown).

Since we found that serum GDF-15 concentrations were independently associated with liver VCTE-derived kPa measurements, and alone explained 21% of the variance in liver VCTE-derived kPa measurements, we next tested whether GDF-15 concentrations and/or T2DM status could predict ≥F2 fibrosis, as determined by the validated VCTE threshold of ≥8.2 kPa [23]. In a model that did not include serum GDF-15 concentrations, T2DM status was associated with ≥F2 fibrosis (Table 3 – model 1). However, when both T2DM status and serum GDF-15 concentrations were added as covariates and ≥F2 fibrosis status was the outcome, only GDF-15 concentration was associated with ≥F2 fibrosis (Table 3 – model 2). Furthermore, in a fully adjusted model where T2DM, GDF-15 concentrations, age, sex, total body fat percentage, e-GFR and AST concentrations were entered as key covariates (identified from multivariable linear regression modelling see Supplementary Table 3) and ≥F2 fibrosis status was the outcome variable, only serum GDF-15 concentrations were associated with ≥F2 fibrosis (Table 3 – model 3). Goodness of fit for the models was tested with Hosmer–Lemeshow tests. A model that only included GDF-15 concentrations as the explanatory variable showed excellent goodness of fit (chi-squared statistic = 2.71, *p* = 0.95). Additionally, as HOMA-IR was significantly higher in NAFLD patients with T2DM compared to those without T2DM and insulin resistance may be an important factor in the relationship between T2DM and liver fibrosis, we explored whether GDF-15 concentrations were associated with liver fibrosis severity, independently of HOMA-IR. In a model where ≥F2 fibrosis status was the outcome and HOMA-IR, T2DM status and GDF-15 concentrations were the explanatory variables, only GDF-15 concentration (and not HOMA-IR or T2DM) was associated with ≥F2 fibrosis [OR = 1.002 (1.001–1.003), *p* = 0.004, for each 1 pg/ml of GDF-15]. Furthermore, we found strikingly similar results for ≥F3 fibrosis status (data not shown but available from the authors).

**Table 3:** Binary logistic regression analysis showing that only serum GDF-15 concentrations and T2DM status were significant independent predictors of a predicted liver fibrosis severity of ≥F2 (as measured by VCTE).

Variables	OR (95% CI)	*P*-value
Model 1		
T2DM status	3.18 (1.3–7.72)	0.01
Model 2		
T2DM status	1.16 (0.39–3.48)	0.79
Serum GDF-15 (pg/ml)	1.002 (1.001–1.003)	0.001
Model 3^a^		
T2DM status	1.30 (0.4–4.2)	0.72
Serum GDF-15 (pg/ml)	1.002 (1.001–1.003)	0.006

Model 1 only contains T2DM status. Note: GDF-15 ORs are for each 1 pg/ml of GDF-15. Dependent variable was liver VCTE measurements <8.2 kPa vs. ≥8.2 kPa (0 and 1, respectively) as a proxy threshold for the identification of ≥F2 fibrosis. Sample size *n* = 93.

*T2DM* type 2 diabetes mellitus, *AST* aspartate aminotransferase, *VCTE* vibration-controlled transient elastography, *GDF-15* growth differentiation factor-15, *e-GFR* estimated glomerular filtration.

^a^Model is adjusted for age, sex, total body fat percentage, e-GFR, and AST concentrations.

**Fig. 2:**
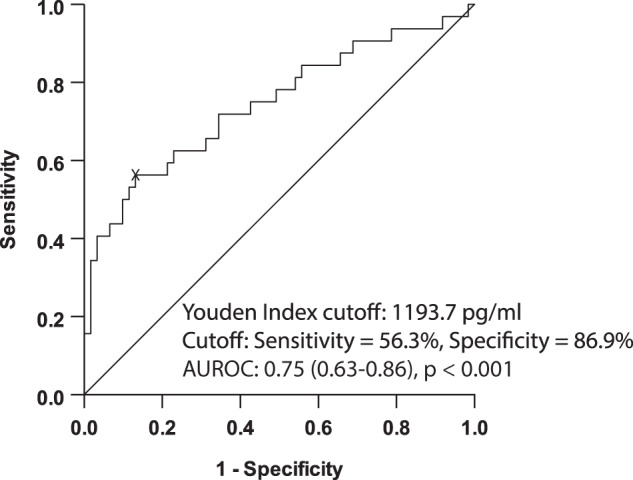
Receiver-operating characteristic curve of serum GDF-15 concentrations for ≥F2 fibrosis (≥8.2 kPa as measured by VCTE). Sample size *n* = 93.

Given this result, we performed ROC curve analysis to assess the ability of GDF-15 concentrations to predict the presence of ≥F2 fibrosis and to identify an optimal GDF-15 concentration cut-off for predicting ≥F2 fibrosis. Accordingly, the AUROC for the prediction of ≥F2 fibrosis was 0.75 (95% CI 0.63–0.86, *p* < 0.001). The Youden index (optimal cut-off) GDF-15 concentration was 1193.7 pg/ml with sensitivity, specificity, positive predictive value (PPV) and negative predictive value (NPV) of 56.3%, 86.9%, 69.2% and 79.1%, respectively (Fig. 2). We then repeated the ROC curve analysis using the higher validated liver kPa threshold for ≥F3 fibrosis (≥9.7 kPa), as the binary outcome. As shown in Supplementary figure 1, these results were remarkably similar to those obtained for ≥F2 fibrosis [AUROC 0.762 (95% CI 0.64–0.89), *p* < 0.0001, optimal cut-off of serum GDF-15 1193.7 pg/ml; sensitivity 63.6%, specificity 83.1%, PPV 53.8% and NPV 88.1%]. In order to assess whether circulating concentrations of GDF-15 were potentially involved in the relationship between T2DM and either ≥F2 fibrosis or ≥F3 fibrosis, we next calculated Sobel test statistics and *p*-values. These data suggested that GDF-15 was potentially involved in the associations between T2DM and ≥F2 fibrosis as well as between T2DM and ≥F3 fibrosis (Sobel test statistics 2.90, *p* = 0.004; and 2.71, *p* = 0.007, for ≥F2 fibrosis and ≥F3 fibrosis, respectively).

### HbA1c levels are independently associated with, and can predict high GDF-15 concentrations in patients with NAFLD

**Fig. 3:**
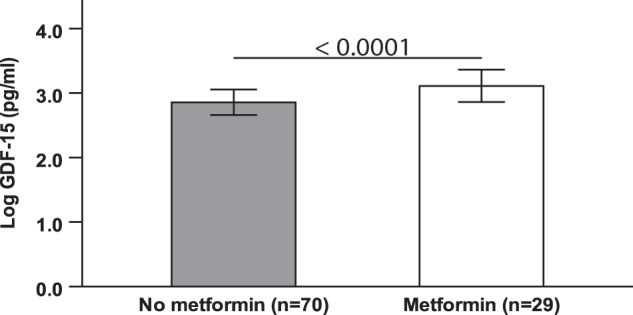
Differences in serum GDF-15 concentrations in patients with NAFLD not receiving vs. receiving metformin treatment. Data are presented as means ± SD.

Given that very little is known regarding the potential regulatory factors of elevated GDF-15 in patients with NAFLD and T2DM, we next looked to identify the factors associated with T2DM that were independently associated with GDF-15 concentrations. GDF-15 concentrations were higher in patients treated with metformin, compared to those not receiving metformin (*p* < 0.0001) (Fig. 3). In univariable analyses, we did not find any significant associations between participant-reported measures of satiety and/or plasma concentrations of satiety hormones with GDF-15 concentrations (Supplementary Table 4). Similarly, none of the measures of satiety (AUC values) was associated with GDF-15 concentrations except for GLP-1 AUC, which was positively associated with GDF-15 concentrations (*r* = 0.30, *p* = 0.003). However, this association was no longer significant after controlling for metformin use (*r* = 0.06, *p* = 0.57). Within the faecal microbiota, there was a greater relative abundance of the Enterobacteriaceae family in the high vs. the low GDF-15 concentration tertile (*q* = 0.003). Similarly, according to univariable correlation analyses, serum GDF-15 concentrations were associated with the abundance of Enterobacteriaceae family (*r* = 0.52, *p* < 0.0001) in faecal samples. However, in multivariable regression modelling, this family of bacteria was not independently associated with GDF-15 concentrations (data not shown).

In regression modelling, the explanatory factors that were independently (all *p* = 0.01 or less) associated with higher GDF-15 concentrations were as follows: higher HbA1c, older age, higher AST, metformin treatment, higher hs-CRP and lower e-GFR (model fit *R*^2^ = 0.60, *p* < 0.00001) (Table 4). Collectively, these factors together explained 60% of the total variance in GDF-15 concentrations. None of the measures of appetite, hunger and/or satiety was independently associated with GDF-15 concentrations (data not shown). As we were able to explain a substantial proportion of the total variance in GDF-15 concentrations within the present cohort, we undertook stepwise linear regression modelling to investigate the proportion of the variance in GDF-15 concentrations that could be explained by each of the aforementioned independent factors. In doing so, we found HbA1c alone explained 29.6% of the total variance in GDF-15 concentrations (Table 4, model 1). The addition of age (model 2) led to a statistically significant increase in *R*^2^ of 0.102 (*p* < 0.001), AST and increase in *R*^2^ of 0.083 (*p* < 0.001) (model 3), metformin use an increase in *R*^2^ of 0.062 (*p* = 0.001) (model 4), hs-CRP an increase in *R*^2^ of 0.03 (*p* = 0.013) (model 5) and e-GFR an increase in *R*^2^ of 0.028 (*p* = 0.015) (model 6). Thus, the addition of each of these independent factors explained a further 10.2% (age), 8.3% (AST), 6.2% (metformin treatment), 3.0% (hs-CRP) and 2.8% (e-GFR), respectively, compared to the 29.6% of the total variance in GDF-15 concentrations, explained by HbA1c alone.

**Table 4:** Multivariable linear regression models explaining variance in serum GDF-15 concentrations.

Independent variables	*R*-square (*R*^2^) of regression model	*R*^2^ change	*p*-value
Model 1	0.296	0.296	<0.00001
HbA1c (mmol/mol)			
Model 2	0.397	0.102	<0.001
HbA1c (mmol/mol) and age (yrs)			
Model 3	0.480	0.083	<0.001
HbA1c (mmol/mol), age (yrs) and AST (IU/l)			
Model 4	0.542	0.062	0.001
HbA1c (mmol/mol), age (yrs), AST (IU/l) and metformin use (yes)			
Model 5	0.572	0.030	0.013
HbA1c (mmol/mol), age (yrs), AST (IU/l), metformin use (yes) and hs-CRP (mg/l)			
Model 6	0.60	0.028	0.015
HbA1c (mmol/mol), age (yrs), AST (IU/l), metformin use (yes), hs-CRP (mg/l) and e-GFR (ml/min/1.73 m^2^)			

Sample size, *n* = 99. In all regression models, the dependent variable was the logarithmically transformed serum GDF-15 concentrations (pg/ml).

NB: *R*-square (*R*^2^ or the coefficient of determination) is a statistical measure in a regression model that determines the proportion of variance in the dependent variable that can be explained by the independent variables.

*AST* aspartate aminotransferase, *hs-CRP* high-sensitivity C-reactive protein, *e-GFR* estimated glomerular filtration rate, *CI* confidence interval.

**Fig. 4:**
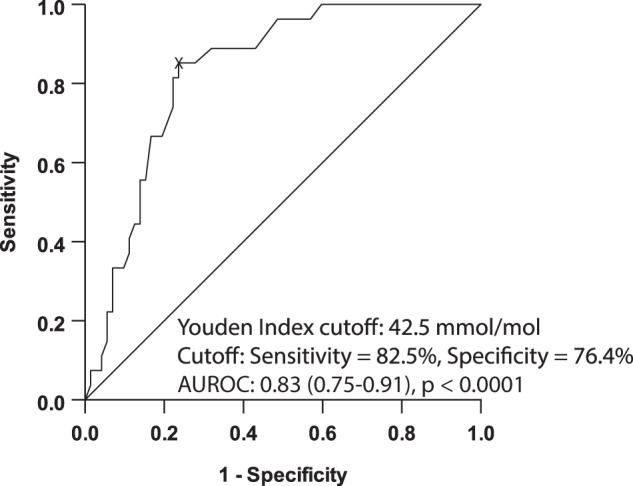
Receiver-operating characteristic curve of HbA1c concentrations for a high serum GDF-15 concentration. State variable was serum GDF-15 concentrations <1193.7 pg/ml vs. ≥1193.7 pg/ml (0 and 1, respectively). Sample size *n* = 99.

We next tested whether increased HbA1c concentration was associated with a high GDF-15 concentration using a GDF-15 threshold of ≥1193.7 pg/ml that we had identified as the optimal cut-off for the prediction of ≥F2 fibrosis (Fig. 2). We carried out binary logistic regression modelling where, in the first model, only HbA1c concentration was entered as a covariate and GDF-15 concentrations were entered as the binary outcome [<1193.7 pg/ml (*n* = 72) vs. ≥1193.7 pg/ml (*n* = 27)]. In this regression model, higher HbA1c was associated GDF-15 concentrations (OR, 1.07; 95% CI 1.0–2.0; *p* < 0.0001) (Supplementary Table 5 – model 1). In the final adjusted model where HbA1c, age, metformin treatment, hs-CRP, AST and e-GFR were entered as covariates, and GDF-15 concentrations were entered as the binary outcome, higher HbA1c, metformin use, higher hs-CRP, higher AST and lower e-GFR were all independently associated with higher GDF-15 concentrations (model 2). Next, we carried out a ROC curve analysis to assess whether HbA1c predicted high GDF-15 concentrations. The AUROC for the prediction of GDF-15 concentrations was 0.83 (95% CI 0.75–0.91) and the optimal cut-off HbA1c concentration was 42.5 mmol/mol (sensitivity, specificity, PPV and NPV were 85.2%, 76.4%, 57.5% and 93.2%, respectively) for the prediction of GDF-15 concentrations (Fig. 4).

## Discussion

The novel findings in this study are that in patients with NAFLD, HbA1c concentrations explain a large proportion (~30%) of the variance in GDF-15 concentrations and that circulating concentration of GDF-15 may be involved in the known association between T2DM and liver fibrosis. This study provides the most in-depth investigation of factors independently associated with serum GDF-15 concentrations in patients with, and without, T2DM who have NAFLD, and also demonstrates that older age, higher HbA1c, higher AST, higher hs-CRP, lower e-GFR and metformin use (but not gut microbiota composition, adipokines or measures of satiety) were all independently associated with higher serum GDF-15 concentrations. Collectively, these factors explained a large proportion (60%) of the total variance in GDF-15 concentrations. Moreover, ROC curve analysis also confirmed that HbA1c was a good predictor of high GDF-15 concentrations.

Our findings that a serum GDF-15 concentration of 1193.7 pg/ml was the optimal threshold for predicting ≥F3 fibrosis are consistent with other recent work carried out in an Asian NAFLD cohort where a serum GDF-15 concentration of 1520 pg/ml was found to be the optimal threshold for predicting histologically proven advanced fibrosis (≥F3 liver fibrosis) [13]. Furthermore, data from a recent large multicentre transcriptomics study identified hepatic GDF-15 expression as a key factor strongly and positively associated with liver fibrosis severity in patients with NAFLD [10]. In addition to liver fibrosis, our findings that GDF-15 concentrations were significantly increased in patients with NAFLD and T2DM compared to those without T2DM, are consistent with various previous studies indicating that GDF-15 concentrations are increased in patients with T2DM [7, 26, 27].

Importantly, a strength of our study is the significant proportion of patients with T2DM and NAFLD (42.4%) and the wide range of HbA1c concentrations (27.0 to 95.0 mmol/mol). Considering the chronic nature of NAFLD and that GDF-15 expression is stress-inducible, it is likely that a range of factors commonly associated with T2DM and/or liver fibrosis is also associated with increasing GDF-15 concentrations. Considering this, we investigated multiple factors and found that, increased HbA1c concentrations were most strongly associated with increased GDF-15 concentrations and that HbA1c concentrations were a good predictor of high (≥1193.7 pg/ml) GDF-15 concentrations with an AUROC of 0.83 (95% CI 0.75–0.91). Interestingly, the optimal cut-off of HbA1c concentration for predicting high GDF-15 concentrations was 42.5 mmol/mol, which is remarkably similar to the threshold for diagnosing pre-diabetes in patients. In addition to this, we found that HbA1c concentrations explained a large proportion (~30%) of the total variance in circulating GDF-15 and were a good predictor of high GDF-15 concentrations, supporting our findings that GDF-15 concentrations may be involved in the relationship between T2DM and liver fibrosis. These findings could suggest that chronic hyperglycaemia has a role in increasing the circulating concentrations of GDF-15 in patients with both NAFLD and T2DM. Interestingly, administration of a high glucose load resulted in a rise in serum GDF-15 concentrations in both non-obese and obese individuals suggesting a potential direct role of hyperglycaemia on increased circulating GDF-15 concentrations [28, 29]. Conversely, given the increased NAFLD severity observed in patients with coexisting T2DM, it is also likely that an increased hepatic expression of GDF-15, due to hepatic inflammation and/or fibrosis and exacerbated by the presence of T2DM, also contributes to elevations in circulating concentrations of GDF-15. Furthermore, whilst a growing body of evidence suggests that GDF-15 may have a pro-fibrogenic role within the liver [13, 14], others have found GDF-15 to be protective and to ameliorate NASH and other metabolic disorders in mice [30, 31]. Consequently, further work should be carried out to elucidate the functional role of GDF-15 in liver fibrosis in NAFLD. Additionally, research should look to explore the potentially additive effects of hyperglycaemia, and other factors involved in the T2DM milieu, on the expression and circulating concentrations of GDF-15.

Recent pre-clinical and clinical studies of T2DM suggest that GDF-15 expression is also increased by oral metformin treatment and that the beneficial effects of metformin on weight loss (and associated hyperglycaemia) may be mediated by metformin-induced GDF-15 acting centrally to suppress appetite [18, 19]. In our study, we show for the first time that metformin treatment is associated with higher GDF-15 concentrations in patients with T2DM and NAFLD, and this association is independent of potential confounding factors. However, in contrast to HbA1c, we found that metformin treatment explained very little of the total variance in GDF-15 concentrations within our cohort (6% vs ~30% for HbA1c). Similarly, we found that of the investigated inflammatory markers, only increased hs-CRP was independently associated with increased GDF-15 concentrations. However, similar to metformin treatment, hs-CRP only explained a small proportion (3%) of the total variance in GDF-15 concentrations. Furthermore, we found that changes in the faecal microbiota, circulating LPS and adipokine concentrations and patient-reported appetite, hunger and/or satiety were not independently associated with serum GDF-15 concentrations.

Our study has some limitations. Firstly, we used the validated VCTE-derived threshold of ≥8.2 kPa and ≥9.7 kPa as proxies for the identification of patients with ≥F2 and ≥F3 fibrosis, respectively [23], instead of liver histology diagnosed fibrosis. That said, growing evidence indicates that liver VCTE has good diagnostic accuracy for the identification of liver fibrosis in patients with NAFLD [32]. Furthermore, a recent large study validated the use of a liver VCTE threshold of ≥8.2 kPa and ≥9.7 kPa as good diagnostic thresholds for identifying ≥F2 (AUROC; 0.77, 95% CI 0.72–0.82) and ≥F3 (AUROC; 0.80, 95% CI 0.75–0.84) fibrosis validated by histology [23]. Our study also utilised a relatively small cohort and further work should also be carried out in larger cohorts with access to liver biopsy data to further investigate the role of circulating GDF-15 in the relationship between T2DM and liver fibrosis. Whilst evidence does suggest that GDF-15 may have a pro-fibrogenic role within the liver and we found that HbA1c explains almost 30% of the variance in GDF-15 concentrations, our findings showing that GDF-15 may be involved in the known association between T2DM and liver fibrosis should be interpreted with caution. With the current study design, we are unable to address causation and we suggest that further work is required to explore the functional role of GDF-15 in the known association between T2DM and liver fibrosis in patients with NAFLD. We also did not collect data on metformin treatment dosage or duration of treatment within the current cohort and we are not able to investigate whether dose-or time-dependent effects exist between metformin use and serum GDF-15 concentrations.

In conclusion, in patients with NAFLD and T2DM, GDF-15 concentrations predicted both ≥F2 and ≥F3 liver VCTE determined fibrosis, and GDF-15 concentrations may be involved in the association between T2DM and liver fibrosis in NAFLD. Furthermore, we explained a large proportion (~60%) of the variance in GDF-15 concentrations and found that HbA1c alone explained almost 30% of that variance. Further investigations are warranted to establish the causal or consequential role of GDF-15 in liver fibrosis and to further explore the potential implementation of circulating GDF-15 as a biomarker for liver fibrosis in patients with NAFLD and T2DM.

## Supplementary information

Supplementary material
