# Supplementary material for: Growth differentiation factor-15 and the association between type 2 diabetes and liver fibrosis in NAFLD
Source: Nutr Diabetes. 2021 Oct 18;11:32. doi: 10.1038/s41387-021-00170-3 (PMC8523689; doi:10.1038/s41387-021-00170-3)
Supplement: Supplementary file 1 — Supplementary material [file 41387_2021_170_MOESM1_ESM.docx]

**SUPPLEMENTARY MATERIALS AND METHODS**

**Liver fat measurements**

Proton magnetic resonance spectroscopy was used for the quantification of intra-hepatic fat content. Briefly, three 20 x 20 x 20-mm^3^ spectroscopic volumes of interest were positioned within segments 3 (inferior sub-segment of the lateral segment), 5 (inferior sub-segment of the anterior segment) and 8 (superior sub-segment of the anterior segment) of the liver, avoiding major blood vessels, intra-hepatic bile ducts, and the lateral margins of the liver. Liver fat percentage was then calculated as the mean value of the liver fat percentages in the three aforementioned liver segments.

**Assessment of hepatic mitochondrial function (13C-ketoisocaproate breath test)**

The 13C-ketoisocaproate breath test (13C-KICA BT) protocol was undertaken in patients with NAFLD as previously described (1). Subjects refrained from alcohol and had fasted overnight for at least 12 h prior to each test. All subjects were at rest for the duration of the study and remained fasted throughout. On the morning of the study, to standardize CO_2_ production, subjects were asked to lie down on a bed and carbon dioxide produced (VCO_2_) at rest was measured by indirect calorimetry (GEM Nutrition, UK) for 25 min prior to the start of the 13C-KICA BT. Paired breath samples for baseline measurement of isotopic abundance were collected from each subject exhaling directly into 12-ml Exetainer breath tubes (LABCO Ltd, High Wycombe, UK) via straws. Each subject then ingested a solution containing 1 mg kg^−1^ body weight of 2-keto-[1-^13^C]-isocaproic acid (99% -^13^C; Cambridge Isotopes, USA) along with 20 mg kg^−1^ body weight of L-leucine (Sigma)in 200 ml of water. Further paired breath samples were then collected every 10 min for 60 min. The enrichment of ^13^C (atom % excess) in CO_2_ in breath at time (t) was calculated from the ^13^C abundance measurements by continuous flow isotope ratio mass spectrometry (CF-IRMS, ABCA System, SERCON, Crewe, UK) and used with a measure of the total CO_2_ production to calculate the cumulative percent ^13^C-dose recovered over 1 h (cPDR over 1 h). The cPDR over 1 h has been previously validated as a measure of HMF (2-4).

**Liver fibrosis biomarkers**

Tissue inhibitor of matrix metalloproteinase-1 (TIMP-1) and hyaluronic acid (HA) have been analysed using ELISA Kits (R&D) read on Luminex 200 (Bio-plex 200 - Bio-Rad, Watford, UK). The Procollagen-III N-terminal Propeptide (PIIINP) assay has been performed with a UniQ radioimmunoassay kit supplied by Orion Diagnostica (Product no.68570). Platelet counts were obtained using a Sysmex XN10 analyser and impedance platelet count. We have generated three different liver fibrosis scores, namely: ELF (5), APRI (6) and FIB-4 (7, 8). The first of these scores has been calculated using measurement of HA, PIIINP and TIMP-1 (5). APRI scores were calculated as previously described with an upper limit of normal for AST of 40 IU/L (6). FIB-4 scores were obtained as previously described (7, 8).

**Appetite, hunger and satiety assessment**

Participants were instructed to abstain from alcohol consumption and strenuous physical activity for 2 days prior to the day of the satiety test (9, 10). Participants were offered a free-choice buffet breakfast (comprising yoghurt, bread, butter, cheese, jam, fruit, orange juice and water; approximately 470 kcal (11)) and were instructed to complete this within 15 minutes. Food and drink were weighed before and after the meal and energy intake was calculated accordingly. Before and after breakfast (at -5, 0, 15, 30, 60, 120 and 180 minutes), appetite ratings were assessed using a 100-mm visual analogue scale (12). Venous blood samples were also taken at each time-point and used to measure plasma satiety hormone concentrations (see Methods section in manuscript text. For the assessment of appetite, a satiety visual analogue scale with verbal descriptors expressing the most positive and the most negative ratings positioned at each end of a 100-mm line. Participants were asked to draw a vertical mark across the line corresponding to their feelings from 0 (not hungry at all) to 100 (very hungry). Satiety was measured by the distance from the left end of the line to the mark placed by each participant (12). The area under the curve (AUC) values were calculated using the trapezoidal rule for both participants reported satiety and satiety hormone concentrations and are expressed as (mm/min) and (pmol/mol/min), respectively.

**Gut microbiota analyses – DNA extraction, sequencing, bioinformatics, and statistical analysis**

Initial quality-filtering of the reads was conducted with the Illumina Software, yielding an average of 169,858 pass-filter reads per sample. with the FastQC software (<http://www.bioinformatics.babraham.ac.uk/publications.html>), and reads were trimmed to 220 bp (R1) and 200 bp (R2) with the FASTX-Toolkit (<http://hannonlab.cshl.edu/fastx_toolkit/>). The reads were merged with the merge-Illumina-pairs application (with P = 0.03, enforced Q30 check, perfect matching to primers, which are removed by the software, and otherwise default settings including no ambiguous nucleotides allowed) (13). For all samples but two, a subset of 26,000 reads was randomly selected using Mothur (14) to avoid large disparities in the number of sequences. Subsequently, the UPARSE pipeline implemented in USEARCH (15) was used to further process the sequences. Amplicon sequence variants (ASVs) were identified using UNOISE3 (16). Taxonomic prediction was performed using the *nbc_tax* function (17), an implementation of the RDP Naive Bayesian Classifier algorithm (18). Raw sequences are available in the SRA database (PRJNA559052). For the statistical analysis of the gut microbiota, the bacterial taxa with a proportional count across all samples below 0.01% and/or present in less than 35% of the patients were removed. Bacterial taxa significantly different between tertiles of serum GDF-15 concentrations were identified using the Mann-Whitney U test, with a Benjamini-Hochberg correction for multiple testing – between tertile gut microbiota analyses are presented as a q-value, which is adjusted for the false discovery rate (i.e. a FDR cut-off of q = 0.05 was considered to be statistically significant). Between tertile gut microbiota analyses were performed in R statistical software.

**SUPPLEMENTARY FIGURE**

**
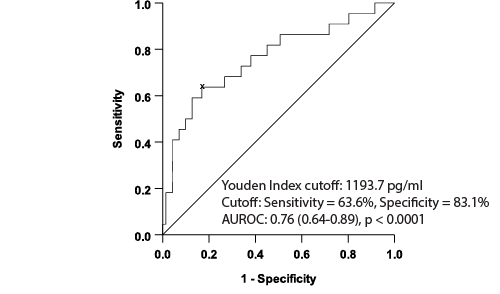
**

**Supplementary figure 1** – **Receiver-operating characteristic curve of serum GDF-15 concentrations for ≥F3 fibrosis (≥9.7 kPa as measured by validated VCTE threshold).** State variable was VCTE derived kPa measurements <9.7 kPa vs. **≥**9.7 kPa (0 and 1 respectively). Sample size n = 93.

**SUPPLEMENTARY TABLES**

**Supplementary table 1 – Measures of appetite and satiety stratified by T2DM status.**

| **Variables** | **Without T2DM**  **(n = 56)** | **With T2DM**  **(n = 42)** | ***p*-value** |
| --- | --- | --- | --- |
| Hunger (mm) | 40.0 ± 23.5 | 41.7 ± 22.7 | 0.85 |
| Fullness (mm) | 31.0 (38) | 21.0 (40.0) | 0.41 |
| Mean satiety (mm) | 51.7 ± 11.1 | 51.6 ± 11.9 | 0.83 |
| Ghrelin (pg/ml) | 94.9 (76.8) | 60.2 (60.1) | 0.001 |
| GIP (pg/ml) | 44.2 (34.6) | 47.4 (34.1) | 0.38 |
| GLP-1 (pg/ml) | 151.6 (47.7) | 181.4 (95) | 0.009 |
| PYY (pg/ml) | 110.0 (69.9) | 128.6 (91.4) | 0.1 |
| PP (pg/ml) | 46.9 (63.1) | 72.6 (93.6) | 0.08 |
| Total calories consumed (kcal) | 285.0 (82.0) | 256.7 (107.0) | 0.08 |
| Hunger (mm/min) | 35.8 ± 16.4 | 40.1 ± 19.5 | 0.24 |
| Fullness (mm/min) | 57.7 (26.1) | 53.4 (29.1) | 0.06 |
| Mean satiety (mm/min) | 61.3 (14.4) | 62.6 (10.6) | 0.5 |
| AUC Ghrelin (pg/ml/min) | 69.3 (66.6) | 55.8 (59.4) | 0.02 |
| AUC GIP (pg/ml/min) | 173.6 (90.4) | 177.7 (110.6) | 0.59 |
| AUC GLP-1 (pg/ml/min) | 161.8 (44.8) | 205.5 (85.5) | <0.0001 |
| AUC PYY (pg/ml/min) | 105.0 (63.2) | 122.0 (83.3) | 0.03 |
| AUC PP (pg/ml/min) | 146.0 (139.3) | 239.3 (270.4) | 0.02 |
|  |  |  |  |

Sample size, n=98. Data for satiety hormones was available for: Without T2DM ≥52 and with T2DM ≥38. Data presented as mean ± SD or median (inter-quartile range). *Abbreviations:* T2DM: type 2 diabetes mellitus, GIP: gastric inhibitory polypeptide, GLP-1: glucagon-like peptide, PYY: peptide YY, PP: pancreatic polypeptide

**Supplementary table 2 - Binary logistic regression models showing associations between other liver fibrosis biomarkers and VCTE kPa measurement cut offs for the identification of ≥ F2 and ≥ F3 liver fibrosis.**

| Biomarker | Model | OR (95%CI) | *P*-value |
| --- | --- | --- | --- |
|  |  |  |  |
| **APRI** | 1**a** | 10.95 (1.85-64.77) | 0.008 |
|  |  | 11.81 (1.82-76.6) ^#^ | 0.01 |
|  |  |  |  |
|  | 2**a** | 10.84 (1.78-65.92) | 0.01 |
|  |  | 11.34 (1.75-73.64) ^#^ | 0.01 |
|  |  |  |  |
|  | 3**a** | 11.63 (1.9-73.0) | 0.009 |
|  |  | 11.36 (1.75-73.73) ^#^ | 0.01 |
|  |  |  |  |
| **FIB-4** | 1**b** | 2.73 (1.33-5.59) | 0.006 |
|  |  | 2.23 (1.09-4.55) ^#^ | 0.03 |
|  |  |  |  |
|  | 2**b** | 2.84 (1.16-6.94) | 0.02 |
|  |  | 2.79 (1.1-7.02) ^#^ | 0.03 |
|  |  |  |  |
|  | 3**b** | 2.94 (1.19-7.3) | 0.02 |
|  |  | 2.80 (1.11-7.05) ^#^ | 0.03 |
|  |  |  |  |
| **ELF** | 1**c** | 2.88 (0.82-10.10) | 0.10 |
|  |  | 2.53 (0.65-9.85) ^#^ | 0.18 |
|  |  |  |  |
|  | 2**c** | 2.32 (0.58-9.33) | 0.24 |
|  |  | 2.67 (0.59-12.20) ^#^ | 0.20 |
|  |  |  |  |
|  | 3**c** | 2.37 (0.59-9.60) | 0.22 |
|  |  | 2.68 (0.59-12.26) ^#^ | 0.20 |

Dependent variables were < F2 liver (< 8.2 kPa) vs ≥ F2 liver (≥ 8.2 kPa) or < F3 liver (< 9.7 kPa) vs ≥ F3 liver (≥ 9.7 kPa) the latter of which is denoted as ^#^. Variables within each model are Model 1a-c; biomarker only, Model 2a-c; biomarker and age and Model 3a-c; biomarker, age and sex. For models including either APRI or FIB-4 sample size, n=94. For models including ELF sample size, n=88. *Abbreviations:* APRI: AST to platelet ratio index , FIB-4: Fibrosis-4, ELF: enhanced liver fibrosis

**Supplementary table 3 – Multivariable linear regression model showing associations with liver VCTE kPa measurement and other liver fibrosis biomarkers**

|  |  | **Unstandardised Coefficient(s)** |  |  |
| --- | --- | --- | --- | --- |
| **Outcome variable** | **Independent variables** | **β** | **95% CI** | **p-value** |
|  |  |  |  |  |
| **A -** Liver VCTE measurement |  |  |  |  |
|  | Log GDF-15 (pg/ml) | 0.35 | 0.15-0.56 | 0.001 |
|  | Age (yrs) | -0.002 | -0.01-0.001 | 0.23 |
|  | Sex (F vs. M) | 0.04 | -0.09-0.16 | 0.54 |
|  | Total body fat (%) | 0.001 | -0.01-0.01 | 0.91 |
|  | T2DM status (yes vs.no) | 0.009 | -0.08-0.09 | 0.83 |
|  | e-GFR (ml/min/1.73 m^2^) | 0.003 | -0.002-0.01 | 0.27 |
|  | AST (IU/l) | 0.0004 | -0.001-0.002 | 0.67 |
|  |  |  |  |  |
| **B -** ELF |  |  |  |  |
|  | Log GDF-15 (pg/ml) | 0.50 | 0.09-0.9 | 0.017 |
|  | Age (yrs) | 0.01 | 0.004-0.02 | 0.001 |
|  | Sex (F vs. M) | -0.07 | -0.31-0.17 | 0.54 |
|  | Total body fat (%) | 0.003 | -0.013-0.02 | 0.68 |
|  | T2DM status (yes vs.no) | -0.05 | -0.22-0.12 | 0.55 |
|  | e-GFR (ml/min/1.73 m^2^) | 0.003 | -0.01-0.01 | 0.57 |
|  | AST (IU/l) | 0.005 | 0.001-0.01 | 0.019 |
|  |  |  |  |  |
| **C -** FIB-4 |  |  |  |  |
|  | Log GDF-15 (pg/ml) | 0.54 | 0.32-0.75 | <0.0001 |
|  | Sex (F vs. M) | -0.01 | -0.15-0.14 | 0.92 |
|  | Total body fat (%) | 0.002 | -0.008-0.01 | 0.68 |
|  | T2DM status (yes vs.no) | -0.06 | -0.16-0.04 | 0.25 |
|  | e-GFR (ml/min/1.73 m^2^) | -0.003 | -0.008-0.002 | 0.28 |
|  |  |  |  |  |
| **D -** APRI |  |  |  |  |
|  | Log GDF-15 (pg/ml) | 0.59 | 0.31-0.87 | <0.0001 |
|  | Age (yrs) | -0.004 | -0.008-0.001 | 0.10 |
|  | Sex (F vs. M) | -0.11 | -0.29-0.07 | 0.23 |
|  | Total body fat (%) | 0.01 | -0.01-0.02 | 0.33 |
|  | T2DM status (yes vs.no ) | -0.05 | -0.18-0.08 | 0.43 |
|  | e-GFR (ml/min/1.73 m^2^) | -0.002 | -0.01-0.01 | 0.63 |

**A**) Sample size, n=93. Logarithmically transformed liver VCTE measurements (expressed as kPa) was included as the outcome variable. R^2^ model = 0.261 (P = 0.001). Stepwise regression analysis also identified that (log) GDF-15 concentration explained 21% of the variance in liver VCTE-derived kPa measurement (N.B. the total variance explained by the above regression model was 26.1%). **B**) Sample size, n=85. ELF scores was included as the outcome variable. R^2^ model = 0.425 (P = <0.0001). Stepwise regression analysis also identified that (log) GDF-15 concentrations explained 30% of the variance in ELF scores. (N.B. the total variance explained by the above regression model was 42.5%). **C**) Sample size, n=88. Logarithmically transformed FIB-4 scores was included as the outcome variable. R^2^ model = 0.345 (P = <0.0001). Stepwise regression analysis also identified that (log) GDF-15 concentrations explained 31.4% of the variance in FIB-4 scores. (N.B. the total variance explained by the above regression model was 34.5%). **D**) Sample size, n=88. APRI scores was included as the outcome variable. R^2^ model = 0.249 (P = 0.001). Stepwise regression analysis also identified that (log) GDF-15 concentrations explained 19.1% of the variance in APRI scores. (N.B. the total variance explained by the above regression model was 24.9%). Note - AST was not included when APRI was the outcome variable and AST and age were not included when FIB-4 was the outcome variable given that these factors are involved in the generation of these liver fibrosis biomarkers. *Abbreviations:* T2DM: type 2 diabetes mellitus, AST: aspartate aminotransferase, VCTE: vibration-controlled transient elastography, APRI: AST to platelet ratio index , FIB-4: Fibrosis-4, ELF: enhanced liver fibrosis, GDF-15; growth differentiation factor-15 and e-GFR: estimated glomerular filtration rate.

**Supplementary table 4 – Univariable associations between serum GDF-15 concentrations and measures of appetite and satiety.**

| **Variables** | **Correlation coefficient(s)** | ***p*- value** |
| --- | --- | --- |
|  |  |  |
| Ghrelin (pg/ml) ^a^ | -0.20 | 0.052 |
| GIP (pg/ml) ^a^ | 0.16 | 0.14 |
| GLP-1 (pg/ml) ^a^ | 0.18 | 0.10 |
| PYY (pmol/mol) ^a^ | 0.1 | 0.36 |
| PP (pg/ml) ^a^ | 0.16 | 0.14 |
| Hunger (mm) | -0.15 | 0.13 |
| Fullness (mm) ^a^ | -0.10 | 0.32 |
| Mean satiety (mm) | 0.02 | 0.88 |
| Total calories consumed (kcal) | -0.12 | 0.26 |
| AUC Ghrelin (pg/ml/min) | -0.18 | 0.09 |
| AUC GIP (pg/ml/min) ^a^ | 0.05 | 0.67 |
| AUC GLP-1 (pg/ml/min) ^a^ | 0.31 | 0.003 |
| AUC PYY (pg/ml/min) ^a^ | 0.09 | 0.40 |
| AUC PP (pg/ml/min) ^a^ | 0.12 | 0.25 |
| Hunger (mm/min) | -0.16 | 0.12 |
| Fullness (mm/min) | 0.09 | 0.40 |
| Mean satiety (mm/min) ^a^ | -0.02 | 0.86 |

Sample size, n= ≥87. ^a^Spearman’s rank correlation coefficients^.^ *Abbreviations: GIP: gastric inhibitory polypeptide, GLP-1: glucagon-like peptide, PYY: peptide YY, PP: pancreatic polypeptide.*

| Variables | OR (95%CI) | *P*-value |
| --- | --- | --- |
| **Model 1** |  |  |
| HbA1c (mmol/mol) | 1.07 (1.03-2.0) | <0.0001 |
|  |  |  |
| **Model 2** |  |  |
| HbA1c (mmol/mol) | 1.08 (1.01-1.15) | 0.018 |
| Age (years) | 1.08 (1.0, 1.2) | 0.05 |
| Metformin treatment (yes vs. no) | 34.2 (3.4-346.7) | 0.003 |
| hs-CRP (mg/l) | 1.65 (1.2-2.23) | 0.001 |
| AST (IU/L) | 1.12 (1.05-1.2) | 0.001 |
| e-GFR (ml/min/1.73 m^2^) | 0.87 (0.77-0.98) | 0.020 |

**Supplementary table 5 – Binary logistic regression analysis showing HbA1c concentrations are an independently associated with increased serum GDF-15 concentration.**

Dependent variable was serum GDF-15 concentration < 1193.7 vs. ≥ 1193.7 pg/ml (0 and 1 respectively). This threshold concentration of GDF-15 was identified as the Youden index optimal threshold for predicting ≥F2 fibrosis (see Figure 2). Sample size, n=99

*Abbreviations:* AST: aspartate aminotransferase, VCTE: vibration-controlled transient elastography, GDF-15; growth differentiation factor-15 and e-GFR: estimated glomerular filtration rate.

**SUPPLEMENTARY REFFERENCES**

1. Afolabi PR, Scorletti E, Smith DE, Almehmadi AA, Calder PC, Byrne CD. The characterisation of hepatic mitochondrial function in patients with non-alcoholic fatty liver disease (NAFLD) using the (13)C-ketoisocaproate breath test. Journal of breath research. 2018;12(4):046002.

2. Berthold HK, Giesen TAH, Gouni-Berthold I. The stable isotope ketoisocaproic acid breath test as a measure of hepatic decarboxylation capacity: a quantitative analysis in normal subjects after oral and intravenous administration. Liver International. 2009;29(9):1356-64.

3. Witschi A, Mossi S, Meyer B, Junker E, Lauterburg BH. Mitochondrial function reflected by the decarboxylation of [13C]ketoisocaproate is impaired in alcoholics. Alcoholism, clinical and experimental research. 1994;18(4):951-5.

4. Lauterburg BH, Grattagliano I, Gmür R, Stalder M, Hildebrand P. Noninvasive assessment of the effect of xenobiotics on mitochondrial function in human beings: studies with acetylsalicylic acid and ethanol with the use of the carbon 13-labeled ketoisocaproate breath test. The Journal of laboratory and clinical medicine. 1995;125(3):378-83.

5. Guha IN, Parkes J, Roderick P, Chattopadhyay D, Cross R, Harris S, et al. Noninvasive markers of fibrosis in nonalcoholic fatty liver disease: Validating the European Liver Fibrosis Panel and exploring simple markers. Hepatology. 2008;47(2):455-60.

6. Loaeza-del-Castillo A, Paz-Pineda F, Oviedo-Cárdenas E, Sánchez-Ávila F, Vargas-Vorácková F. AST to platelet ratio index (APRI) for the noninvasive evaluation of liver fibrosis: Original Article. Annals of Hepatology. 2008;7(4):350-7.

7. Vallet-Pichard A, Mallet V, Nalpas B, Verkarre V, Nalpas A, Dhalluin-Venier V, et al. FIB-4: an inexpensive and accurate marker of fibrosis in HCV infection. comparison with liver biopsy and fibrotest. Hepatology. 2007;46(1):32-6.

8. Lee J, Vali Y, Boursier J, Spijker R, Anstee QM, Bossuyt PM, et al. Prognostic accuracy of FIB-4, NAFLD fibrosis score and APRI for NAFLD-related events: A systematic review. Liver international : official journal of the International Association for the Study of the Liver. 2021;41(2):261-70.

9. Scorletti E, Afolabi PR, Miles EA, Smith DE, Almehmadi A, Alshathry A, et al. Synbiotics Alter Fecal Microbiomes, But Not Liver Fat or Fibrosis, in a Randomized Trial of Patients With Nonalcoholic Fatty Liver Disease. Gastroenterology. 2020;158(6).

10. Scorletti E, Afolabi PR, Miles EA, Smith DE, Almehmadi A, Alshathry A, et al. Design and rationale of the INSYTE study: A randomised, placebo controlled study to test the efficacy of a synbiotic on liver fat, disease biomarkers and intestinal microbiota in non-alcoholic fatty liver disease. Contemporary Clinical Trials. 2018;71:113-23.

11. Gregersen NT, Flint A, Bitz C, Blundell JE, Raben A, Astrup A. Reproducibility and power of ad libitum energy intake assessed by repeated single meals. Am J Clin Nutr. 2008;87(5):1277-81.

12. Flint A, Raben A, Blundell JE, Astrup A. Reproducibility, power and validity of visual analogue scales in assessment of appetite sensations in single test meal studies. International Journal of Obesity. 2000;24(1):38-48.

13. Eren AM, Vineis JH, Morrison HG, Sogin ML. A filtering method to generate high quality short reads using illumina paired-end technology. PLoS One. 2013;8(6):e66643.

14. Schloss PD, Westcott SL, Ryabin T, Hall JR, Hartmann M, Hollister EB, et al. Introducing mothur: open-source, platform-independent, community-supported software for describing and comparing microbial communities. Appl Environ Microbiol. 2009;75(23):7537-41.

15. Edgar RC. UPARSE: highly accurate OTU sequences from microbial amplicon reads. Nat Methods. 2013;10(10):996-8.

16. Edgar RC. UNOISE2: improved error-correction for Illumina 16S and ITS amplicon sequencing. bioRxiv. 2016:081257.

17. Edgar RC. Accuracy of taxonomy prediction for 16S rRNA and fungal ITS sequences. PeerJ. 2018;6:e4652.

18. Wang Q, Garrity GM, Tiedje JM, Cole JR. Naive Bayesian classifier for rapid assignment of rRNA sequences into the new bacterial taxonomy. Appl Environ Microbiol. 2007;73(16):5261-7.
